# Solitary fibrous tumor/hemangiopericytoma in the cerebellopontine angle mimicking vestibular schwannoma: A case report and literature review

**DOI:** 10.1097/MD.0000000000019651

**Published:** 2020-03-27

**Authors:** Xi Yue, Jie Huang, Yaqi Zhu, Yong Du

**Affiliations:** Department of Radiology, Affiliated Hospital of North Sichuan Medical College, Sichuan Province, China.

**Keywords:** cerebellopontine angle, hemangiopericytoma, internal auditory canal, solitary fibrous tumor

## Abstract

**Rationale:**

Intracranial solitary fibrous tumors (SFTs) and hemangiopericytomas (HPCs) are rare spindle cell tumors of mesenchymal origin that include benign and malignant neoplasms.

**Patient concerns:**

We present a 66-year-old male with a 5-year history of headache and dizziness, with left progressive sensorineural hearing loss over 1 month.

**Diagnoses:**

WHO grade II SFT/HPC originating from the internal auditory canal in the left cerebellopontine angle.

**Interventions:**

surgical resection.

**Outcomes:**

No local recurrence or metastases were observed in the follow-up 3 months after the surgery.

**Lessons:**

Intracranial SFTs/HPCs are rare mesenchymal neoplasms that are challenging to manage. If the imaging characteristics of tumor are not typical, clinicians should depend on tissue biopsy and immunohistochemistry to make a definitive diagnosis.

## 1 Introduction

Solitary fibrous tumors (SFTs) and hemangiopericytomas (HPCs) are rare spindle cell tumors of mesenchymal origin that account for less than 2% of all soft tissue masses.^[1]^ In recent years, studies have found that solitary fibrous tumors and hemangiopericytomas of the central nervous system (CNS) have overlapping pathological and immunohistochemistry features, including occurring in the neuraxis, inversions at 12q13, overexpression of the NAB2-STAT6 gene fusion.^[2,3]^ For this reason, the 2016 CNS WHO has created the combined term SFT /HPC to describe such lesions and divided these tumors into 3 categories according to their tumor heterogeneity.^[4]^ The first SFT/HPC involving CNS was reported in 1996 by Carneiro et al^[5]^ CNS SFTs/HPCs account for only 0.09% of all meningeal tumors, while intracranial SFTs/HPCs are extremely rare.^[6]^ Intracranial SFTs/HPCs are extra-axially located and develop from the meninges.^[7]^ Intracranial SFTs/HPCs in the cerebellopontine angle (CPA) are frequently misdiagnosed as meningiomas or schwannomas.^[8]^ We report a case of a benign SFT/HPC in the CPA mimicking vestibular schwannoma (VS). Furthermore, we discuss the differential diagnosis of intracranial masses in the CPA and how to distinguish them based on imaging characteristics.

## 2 Case presentation

**Figure 1:**
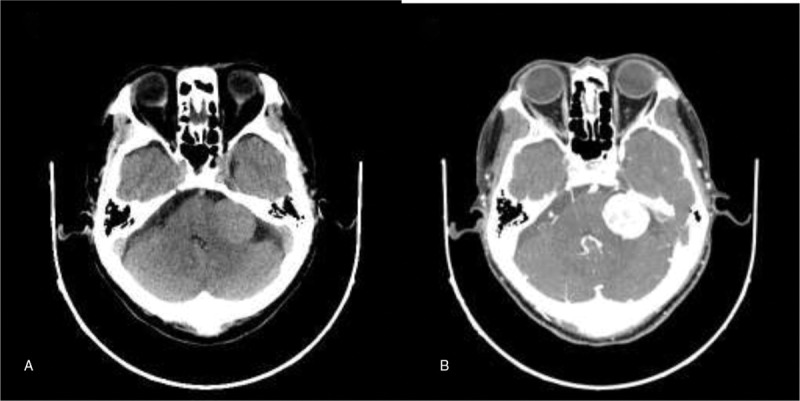
(A) Axial head CT revealed a 3.0 × 2.7 cm well-defined slightly hyperdense mass without peritumoral edema in the left CPA. (B) On enhanced CT, the mass was homogeneously enhancing, extending into IAC. CPA = cerebellopontine angle, CT = computed tomography, IAC = internal auditory canal.

**Figure 2:**
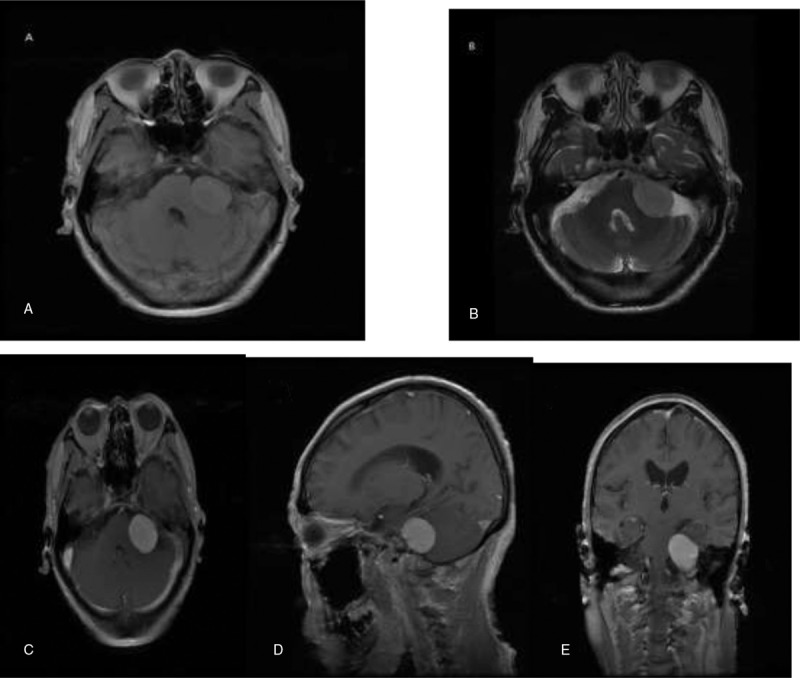
The brain MRI demonstrated a 2.4 × 2.5 × 2.6 cm ovoid mass in the left CPA. The tumor showed intermediate-slightly increased signal intensity in T1WI (A) and slightly increased signal intensity in T2WI (B). Axial (C), sagittal (D), and coronal (E) enhanced T1WI showed homogeneous and strong enhancing tumor, extending into the enlarged IAC and closely connected to the vestibulocochlear nerve. CPA = cerebellopontine angle, IAC = internal auditory canal, MRI = magnetic resonance imaging.

A 66-year-old male presented to neurology outpatient department with a 5-year history of headache and dizziness, and 1 month ago he had left progressive sensorineural hearing loss. His vestibular testing was positive. Computed tomography (CT) of the head revealed a 3.0 × 2.7 cm well-defined slightly hyperdense mass without peritumoral edema in the left CPA. On enhanced CT, the mass was homogeneously enhancing, extending into the enlarged internal auditory canal (IAC). Given the CT characteristics, a provisional diagnosis of a VS was made (Fig. 1A and B). The brain magnetic resonance imaging (MRI) demonstrated a 2.4 × 2.5 × 2.6 cm ovoid mass in the left CPA. The tumor showed intermediate-slightly increased signal intensity in T1-weighted image (T1WI) and slightly increased signal intensity in T2-weighted image (T2WI). Diffusion-weighted imaging (DWI) did not reveal areas with diffusion restriction. On enhanced T1WI, the tumor showed homogeneous and strong enhancement, extending into the enlarged IAC and closely connected to the vestibulocochlear nerve. Given the MRI characteristics, a provisional diagnosis of a VS was made (Fig. 2A, B, C, D, and E).

**Figure 3:**
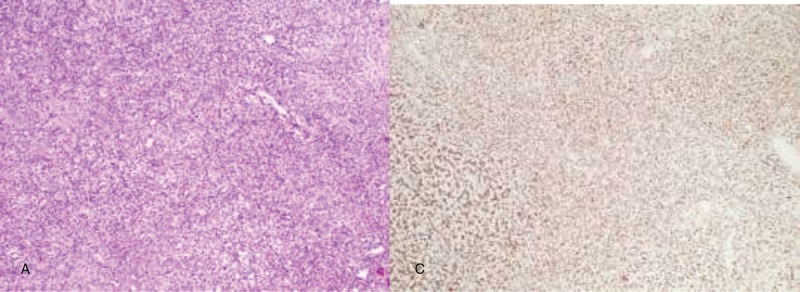
The brain MRIdemonstrated a 2.4 × 2.5 × 2.6 cm ovoid mass in the left CPA. The tumor showed intermediate-slightly increased signal intensity in T1WI (A) and slightly increased signal intensity in T2WI (B). Axial (C), sagittal (D), and coronal (E) enhanced T1WI showed homogeneous and strong enhancing tumor, extending into the enlarged IAC and closely connected to the vestibulocochlear nerve. CPA = cerebellopontine angle, IAC = internal auditory canal, MRI = magnetic resonance imaging.

A surgical resection of the tumor was performed. Surgical findings showed a 3 cm tough and well-vascularized tumor originating from the IAC in the left CPA. The tumor obviously compressed the facial nerve and trigeminal nerve, and adhered to the facial nerve so closely that cannot be completely separated. Further, a biopsy was performed following the tumor resection. Cells were positive for STAT-6, CD34, BCL-2, Ki-67 (10%), and Vimentin. Cells were negative for CK, EMA, S-100, GFAP, SMA, olig-2. With these results, a diagnosis of WHO grade II SFT/HPC was made (Fig. 3A and B). The patient recovered and was discharged from the hospital. There was no evidence of local recurrence or metastases in the follow-up 3 months after the surgery. However, in this case, due to the short follow-up, the prognosis was uncertain, and the patient was advised to return every 3 months for a further follow-up.

## 3 Discussion

Since the first SFT/HPC described by Klemperer et al in 1931 as a pleural tumor,^[9]^ various extra-pleural occurrences of these tumors have been reported.^[10]^ Intracranial SFTs/HPCs are rare mesenchymal neoplasms and constitute a heterogeneous group of rare spindle cell tumors that include benign and malignant neoplasms.^[11]^ Intracranial SFTs/HPCs usually occur in the fifth decade, but without significant gender predilection.^[12]^ When the tumors become large enough or infringe into the important functional areas, patients will show clinical symptoms including episodic headaches, dizziness, gait imbalance, sensory disturbance, hemiplegic paralysis, and epileptic seizure.^[8,12]^

Radiologic features can be helpful in predicting tumor pathology, while pathological examination remains the gold standard in its diagnosis and pathological grading. However, no imaging modality correlates perfectly with any diagnosis. The signal intensity in T2WI of SFTs/HPCs’ solid portions can be helpful to evaluate the pathological grade. For WHO grade I, a majority of the cases show intermediate-low signal intensity in T2WI. A minority of the cases are made of 2 obviously different signal intensity areas in T2WI.^[13]^ T2-hypointense areas represent fibrosis components that show intense contrast enhancement, while T2 iso- or hyperintense areas represent hypercellular components that show moderate heterogeneous enhancement. The combination of these 2 components gives rise to the so-called yin yang sign, which is associated with intracranial SFTs/HPCs.^[14]^ For WHO grade II and III, the tumors usually show intermediate-high signal intensity in T2WI. Tortuous flow-empty vascular shadow is often seen inside or on the surface of these 2 grades tumors, which is essential to differentiate WHO grade I with WHO grade II and III. Besides, the incidence of peritumoral edema in WHO grade II and III is higher than WHO grade I. But in this case, there is no peritumoral edema. SFTs /HPCs in the CPA are frequently misdiagnosed as meningiomas or schwannomas. Radiologically, Meningiomas show dural tail sign and calcification more frequently than SFTs/HPCs; in contrast, SFTs/HPCs show necrosis, cystoid degeneration, and flow-empty actions more frequently than meningiomas. The adjacent bone may help to differentiate meningioma from SFT/HPC. The adjacent bone is often thickened in meningiomas; SFT/HPC, in contrast, can erode the adjacent bone. Schwannoma originates from Schwann cells of the nerve sheath and grows along the nerve sheath. The patient with schwannoma usually has corresponding neurological symptoms. Cystoid degeneration is often seen in these lesions, so MRI usually shows long T1 and long T2 signals. VS is the most common cranial nerve schwannoma. VS is also the most frequent tumor of the CPA and IAC, and arises from Schwann cells around the vestibular nerve and ganglia.^[15]^ The gold standard imaging modality for VS diagnosis is T1WI MR imaging of the IAC with and without contrast. Without contrast, VSs are hyperintense relative to cerebrospinal fluid and are isointense to hypointense relative to gray matter on T1-weighted sequences. VSs obviously enhance with contrast. Cystic VSs can also occur with frequency of 11% to 48%.^[16]^ The expansion of IAC can be used as an important basis for evaluating VS. In this case, CT and MRI both showed that the tumor extended into the enlarged IAC and closely connected to the vestibulocochlear nerve. Besides, the patient had left progressive sensorineural hearing loss. So given the imaging and clinical features of this patient, the tumor is misdiagnosed as VS in this case. If the imaging characteristics of tumor are not typical, an accurate diagnosis is difficult to made. Then, clinicians should depend on tissue biopsy and immunohistochemistry to make a definitive diagnosis. Immunohistochemically, SFTs/HPCs usually show reversed 12q13 and the NAB2–STAT6 fusion gene, while meningiomas are typically reactive for EMA.

Surgery is recommended as the criterion standard of treatment. Complete excision of the mass is clearly superior to incomplete excision, and sub-totally removed SFTs/HPCs may recur or continue to grow.^[17]^ Melone et al^[18]^ found that SFTs/HPCs invading the cerebral venous sinuses significantly recurred earlier. Invasive skull base SFTs/HPCs or those infiltrating deeply into a venous sinus, usually cannot be entirely removed without high risks of postoperative complication. Therefore, the extent of resection is the most powerful prognostic factor for recurrence of SFTs/HPCs.^[19]^ Preoperative embolization, preoperative chemotherapy, and postoperative adjuvant radiotherapy have been performed. Radiotherapy after surgical resection of SFTs/HPCs continues to be controversial. Most neurosurgeons and neuro-oncologists would advocate adjuvant radiotherapy especially if the tumor was not entirely excised. No conclusion can currently be drawn regarding the effectiveness of these measures.^[20]^ In the CNS, the clinical behavior of SFTs/HPCs is particularly aggressive, with recurrence rates of 61% to 76% and metastasis rates of 23% to 64%. Reported 5-year overall survival probabilities vary between 67% and 96% with median overall survival between 7 and 16.2 years.^[21]^

In conclusion, intracranial SFTs/HPCs are rare mesenchymal neoplasms that are challenging to manage. For their rarity and resemblance to other more common brain tumors, they are often poorly recognized. In other words, if the imaging characteristics of tumor is not typical, clinicians should depend on tissue biopsy and immunohistochemistry to make a definitive diagnosis. SFTs/HPCs are associated with a significant risk of recurrence that may reduce overall survival. Even if no researches have showed the effectiveness of these measures, most clinicians advocate that tailored maximal tumor resection upon initial surgery is beneficial and that postoperative adjuvant radiotherapy is useful for SFTs/HPCs displaying grade II or III, even in case of complete resection.

## Author contributions

**Data curation:** Jie Huang, Ya-qi Zhu.

**Supervision:** Yong Du.

**Writing – original draft:** Xi Yue.
